# Association of Urinary Biomarkers and Joint Longitudinal Cognitive-Gait Trajectories: Health ABC Findings

**DOI:** 10.1093/geroni/igaf122.2960

**Published:** 2025-12-31

**Authors:** Aman Shrestha, Michelle Shardell, Chixiang Chen, Stephen L Seliger, Charles Ginsberg, Lindsay Miller, Peggy Cawthon

**Affiliations:** University of Maryland, Baltimore County, Baltimore, Maryland, United States; University of Maryland School of Medicine, Baltimore, Maryland, United States; University of Maryland School of Medicine, Baltimore, Maryland, United States; University of Maryland School of Medicine, Baltimore, Maryland, United States; University of California, San Diego, San Diego, California, United States; University of California, San Diego, San Diego, California, United States; California Pacific Medical Center, San Francisco, California, United States

## Abstract

Emerging urinary biomarkers are unlocking novel insights into the association between kidney tubular injury and cognitive-physical function. Using data from the Health, Aging and Body Composition Study, we examined associations between baseline urinary markers—uromodulin, alpha-1 microglobulin (α1M), amino-terminal propeptide of type-III procollagen (PIIINP), neutrophil gelatinase-associated lipocalin (NGAL), interlukin-18 (IL-18) and kidney injury molecule-1 (KIM-1)—with simultaneous longitudinal cognition-gait trajectories among initially high-functioning older adults. IL-18 and KIM-1 were analyzed in a larger ancillary study (n = 1902), while uromodulin, α1M, PIIINP and NGAL were assessed in a random sub-cohort (n = 502). Grouped-based trajectory analysis of 20-meter usual gait speed (years 3-6, 8, 10) and modified mini-mental state (years 3, 5, 8, 10) identified three groups: Group 1 with consistently high cognitive-physical performance, Group 2 with stable cognition but declining gait, and Group 3 with rapid declines in both domains. In separate models (Model-1, unadjusted), higher α1M, PIIINP, IL-18 and KIM-1 concentrations were associated with poorer trajectories. After adjusting for sociodemographic factors (model-1A), only α1M (p = 0.043) and KIM-1 (p = 0.005) remained significant. Further adjustment for estimated glomerular filtration rate (eGFR), urine albumin-to-creatinine ratio (UACR) and c-reactive protein (CRP) (Model-1B) attenuated all associations, leaving only KIM-1 marginally significant (p = 0.059). In a fully adjusted model that included all urinary markers (Model-2), only KIM remained individually significant (p = 0.012), though the collective effect of six urinary markers was also significant (p = 0.022). Of all urinary biomarkers, KIM-1 emerged as the robust predictor of cognitive-gait decline in understanding the interplay between kidney tubular injury and aging-related functional outcomes.

